# Evaluating the efficacy and tolerability of the oral combination of alpha lipoic acid and vitamin B complex preparation in carpal tunnel syndrome: a single center, randomized, double-blind, placebo-controlled trial

**DOI:** 10.1186/s12883-025-04430-y

**Published:** 2025-12-08

**Authors:** Nur Alya Zainal, Anna Misya’il Abdul Rashid, Adnan Lutfi A Rauf, Abdul Hanif Khan Yusof Khan, Wan Aliaa Wan Sulaiman, Hamidon Basri

**Affiliations:** 1 Department of Medicine, Faculty of Medicine and Health Sciences, Universiti Putra Malaysia, Serdang, Selangor 43400 Malaysia; 2 Department of Neurology, Faculty of Medicine and Health Sciences, Universiti Putra Malaysia, Serdang, Selangor 43400 Malaysia; 3 Department of Neurology, Hospital Sultan Abdul Aziz Shah, Serdang, Selangor 43400 Malaysia

**Keywords:** Vitamin b complex, Alpha lipoic acid, Carpal tunnel syndrome, Electrodiagnosis, Pain measurement, Quality of life, Outcome assessment

## Abstract

**Background:**

Carpal Tunnel Syndrome (CTS) is the most common type of nerve entrapment and can cause significant disability by impairing hand functionality. This study aims to evaluate the efficacy and tolerability of an oral combination of alpha-lipoic acid (ALA) and vitamin B complex in alleviating CTS symptoms and improving the quality of life in patients with mild to moderate CTS.

**Method:**

A total of 70 patients with mild to moderate CTS were enrolled in this study at Hospital Sultan Abdul Aziz Shah, Selangor, Malaysia. Using a double-blind Randomized Controlled Trial (RCT) design, participants were randomly assigned to either the treatment group (*n* = 37) or the placebo group (*n* = 33). The treatment group received two tablets **once** daily containing alpha-lipoic acid (300 mg), methylcobalamin (500 mcg), vitamin B1 (39 mg), and vitamin B6 (8 mg) i.e. Bionerv while placebo group received combination of maltodextrin, microcrystalline cellulose, tricalcium phosphate, silicon gioxide, magnesium stearate; two tablets once daily for 6 months. Patients were evaluated across three visits: baseline (Visit 1), 3 months (Visit 2), and 6 months (Visit 3). During each visit, patients were evaluated using three assessments. The three assessments included three standardized questionnaires: SF-36, VAS, and BCTQ, Nerve Conduction Study (NCS) and Patient-Reported Questionnaire to monitor any side effects.

**Results:**

A significant improvement in VAS scores was observed in the treatment group, indicating better pain control. However, no statistically significant differences were found between the treatment and placebo groups in other outcome measures, including SF-36, BCTQ, and NCS parameters. While improvements in SF-36 and BCTQ scores were noted in the treatment group, these changes did not reach statistical significance and should be interpreted as a positive trend rather than definitive evidence of efficacy. The combination of alpha-lipoic acid and vitamin B complex was well tolerated, with most participants reporting minimal and self-limiting adverse effects. The most common side effects were mild gastrointestinal symptoms, such as abdominal discomfort and diarrhea, which resolved upon discontinuation of drugs.

**Conclusion:**

The oral combination of alpha-lipoic acid and vitamin B complex (Bionerv) demonstrated potential pain relief benefits in patients with mild to moderate CTS and was found to be well tolerated. Future studies with larger sample sizes are recommended to strengthen the findings and provide more comprehensive insights.

**Supplementary Information:**

The online version contains supplementary material available at 10.1186/s12883-025-04430-y.

## Background

Carpal tunnel syndrome (CTS) is the most common form of nerve entrapment neuropathy in the hand, and it has a high global prevalence, including in Malaysia, where an estimated 20–60% of the population is diagnosed with CTS [1, 2]. CTS significantly impairs hand functionality, leading to substantial disability. A study in the United States reported CTS-related disability incidence rates per 100 person-years as 33.2 for work pace or quality changes, 16.3 for lost time, and 20.0 for job changes, emphasizing its impact on occupational productivity [3]. Lost time, defined as workdays missed due to CTS, averaged 28 days in 2015—comparable to fractures and exceeding amputations [4, 5]. The substantial economic and functional burden of CTS underscores the necessity for effective therapeutic strategies to mitigate its impact.

Current treatment options for CTS include surgical decompression for severe cases and multimodal therapy for mild to moderate cases, consisting of conservative treatments such as oral medications (vitamin B complex, analgesia), splinting, physiotherapy, and injections (steroids or platelet-rich plasma) [6]. However, local steroid and anaesthetic injections are costly and invasive, thus not preferred by patients, while long-term analgesia use poses sedative risks [7]. Alpha-lipoic acid (ALA), known for its benefits in diabetic neuropathy, is hypothesized to aid in CTS by promoting nerve regeneration and reducing pain, though its impact on functional outcomes remains limited [8]. Previous studies on alpha-lipoic acid (ALA) have reported mild side effects such as nausea, abdominal discomfort, dizziness, and skin reactions thus it is important also to reassess the tolerability of the ALA and vitamin B complex combination in the local population [9].

Alpha-lipoic acid has shown promise in peripheral nerve disorders and is increasingly explored for carpal tunnel syndrome (CTS) due to its potent antioxidant properties. CTS pathophysiology involves ischemia-reperfusion injury, which elevates oxidative stress and impairs nerve regeneration [10]. ALA mitigates oxidative damage by neutralizing free radicals, restoring antioxidant activity (e.g., vitamins E and C), and boosting glutathione levels essential for tissue repair [11]. Clinical studies and meta-analyses have demonstrated ALA’s efficacy in reducing pain and improving symptoms, particularly in postoperative CTS management ([9, 12]– [13]). However, local data remain limited, highlighting the need for further research in the Malaysian population.

## Methodology

### Study design and participants

This was a double-blind, placebo-controlled randomized controlled trial, from December 2023 to September 2024 in a tertiary center in Malaysia. Relevant national regulatory authorities and ethics committees approved the study protocol (JKEUPM-2022-98) and the protocol was registered with Clinical Trial Gov (NCT06940557) retrospectively. We recruited patients aged 18 years and above who were diagnosed with CTS. The inclusion criteria included anyone with numbness and tingling sensation at the first finger until the radial site of the 4th finger and/or fulfil the electrodiagnostic criteria of mild to moderate CTS, based on the Padua Scale as defined below [14]:

• Mild: abnormal digit/wrist Sensory Nerve Conduction Velocity (CV) and normal Distal Motor Latency (DML).

- Moderate: abnormal digit/wrist Sensory Nerve Conduction Velocity (SNCV) and abnormal Distal Motor Latency (DML).

We excluded patients who were pregnant or breastfeeding, with symptoms of CTS but have normal NCS, taking traditional or complementary medication for CTS or patients with electrodiagnostic criteria of severe or extreme CTS as defined in the Padua scale:

• Severe: Absence of sensory response (SNAP) and abnormal Distal Motor Latency (DML).

- Extreme: Absence of motor (CMAP) and sensory responses (SNAP).

### Study treatment and procedures

The patients were randomized to receive oral combination of alpha lipoic acid and vitamin B; Bionerv (alpha lipoic acid; 300 mg, methylcobalamin; 500mcg, vitamin B1; 39 mg and vitamin B6; 8 mg), 2 tablets once daily or placebo drug (maltodextrin, microcrystalline cellulose, tricalcium phosphate, silicon gioxide, magnesium stearate), 2 tablets once daily for 6 months. Randomization sequence was done by using an online random number generator program using a mixed block randomization technique at a ratio of 1:1. In cases of serious adverse event, unblinding process was incorporated where the identity of the drug was revealed to manage the subject’s condition. This was done by intruding the emergency code key by the researcher. All patients provided a written consent for the study.

During Visit 1, a thorough history and physical examination were performed, and demographic data were collected. Patients then completed three validated, self-administered questionnaires: the Boston Carpal Tunnel Questionnaire (BCTQ), Visual Analogue Scale (VAS), and SF-36. The BCTQ is a disease-specific tool for assessing CTS severity, comprising two subscales: the Symptom Severity Scale (SSS) with 11 items and the Functional Status Scale (FSS) with 8 items. Each item is rated on a 5-point Likert scale (1 = no symptoms/difficulty, 5 = most severe symptoms/greatest difficulty). The total score for each subscale is calculated as the mean of its item scores, resulting in a final SSS and FSS score ranging from 1.0 to 5.0. Higher scores reflect worse symptoms or greater functional impairment. The BCTQ is widely used due to its strong cross-cultural adaptability and proven validity and reliability across multiple languages ([15]– [16]).

The VAS is a visual scale with 0 scored as “no pain” and 10 as “worst pain imaginable” and serves as an excellent tool for pain score with excellent validity and reproducibility ([17]– [18]). The SF-36 is a quality-of-life questionnaire that comprises of 36 questions covering eight domains of health which are; Physical Functioning (PF), Role Limitations due to Physical Health, Role Limitations (RL) due to Emotional Problems (RE), energy/fatigue, emotional well-being, social functioning, pain, and general health (GH) where a higher score indicates a better outcome [19]. After the administration of these questionnaires, they will be subjected for a nerve conduction study (NCS) to determine the severity of CTS. The NCS were performed by the same trained personnel to ensure consistency and accuracy.

The patients were reassessed at Visit 2 (3 months post-treatment) and Visit 3 (6 months post-treatment). During each visit, they underwent physical examination, NCS, and were required to complete the three sets of questionnaires (BCTQ, VAS, and SF-36). They were also required to fill in the form regarding the side effects of the treatments at Visit 2 and Visit 3. The investigators made a phone call and sent reminder message at the 6th and 18th week to ensure compliance, medication adequacy and follow up review. During the study, all participants received standard care, including nighttime wrist splinting and physiotherapy when indicated. Physiotherapy included tendon and nerve gliding exercises and ultrasound therapy, applied equally to both groups to ensure consistent care aside from the intervention.

### Outcomes

The primary outcome was the electrodiagnostic improvement of the median nerve on NCS, assessed between baseline (Visit 1, prior to intervention), and compared to follow-up evaluations at three months (Visit 2) and six months (Visit 3) after the intervention. The NCS parameters that are analysed were: DSL (normal value for DSL < 3.5ms), SNAP (normal value for SNAP > 2.5 µV), CV (normal value for CV > 50 m/s), DML (normal value for DML < 4ms), CMAP (normal value for CMAP > 4.0 mV) [20]. The secondary outcomes measure were the changes in BCTQ, VAS and SF-36 scores between the intervention (visit 1) and at three-months (visit 2) and six- months (visit 3) of treatment as well as side effects of the treatment.

### Statistical analysis

The data analysis was performed using the IBM SPSS Statistics for Windows, Version 27.0 (IBM Corp., Armonk, NY, USA). The descriptive statistics were expressed as numbers and percentages for categorical variables and as mean for numerical variables. Repeated measure ANOVA statistical analysis was used to assess the effect of intervention on the primary and secondary outcomes. The confidence level was set at 0.05 (α = 0.05) and significant finding was if the p-value is less than 0.05 (*p* < 0.05). Data normality was assessed using the skewness test, with normal distribution defined within the range of + 2 to −2. Mauchly’s sphericity test was conducted, with most results achieving *p* > 0.05, indicating that the assumption of sphericity was met. For data that violated sphericity, the Greenhouse-Geisser correction was applied.

## Results

### Baseline demographic

**Fig. 1:**
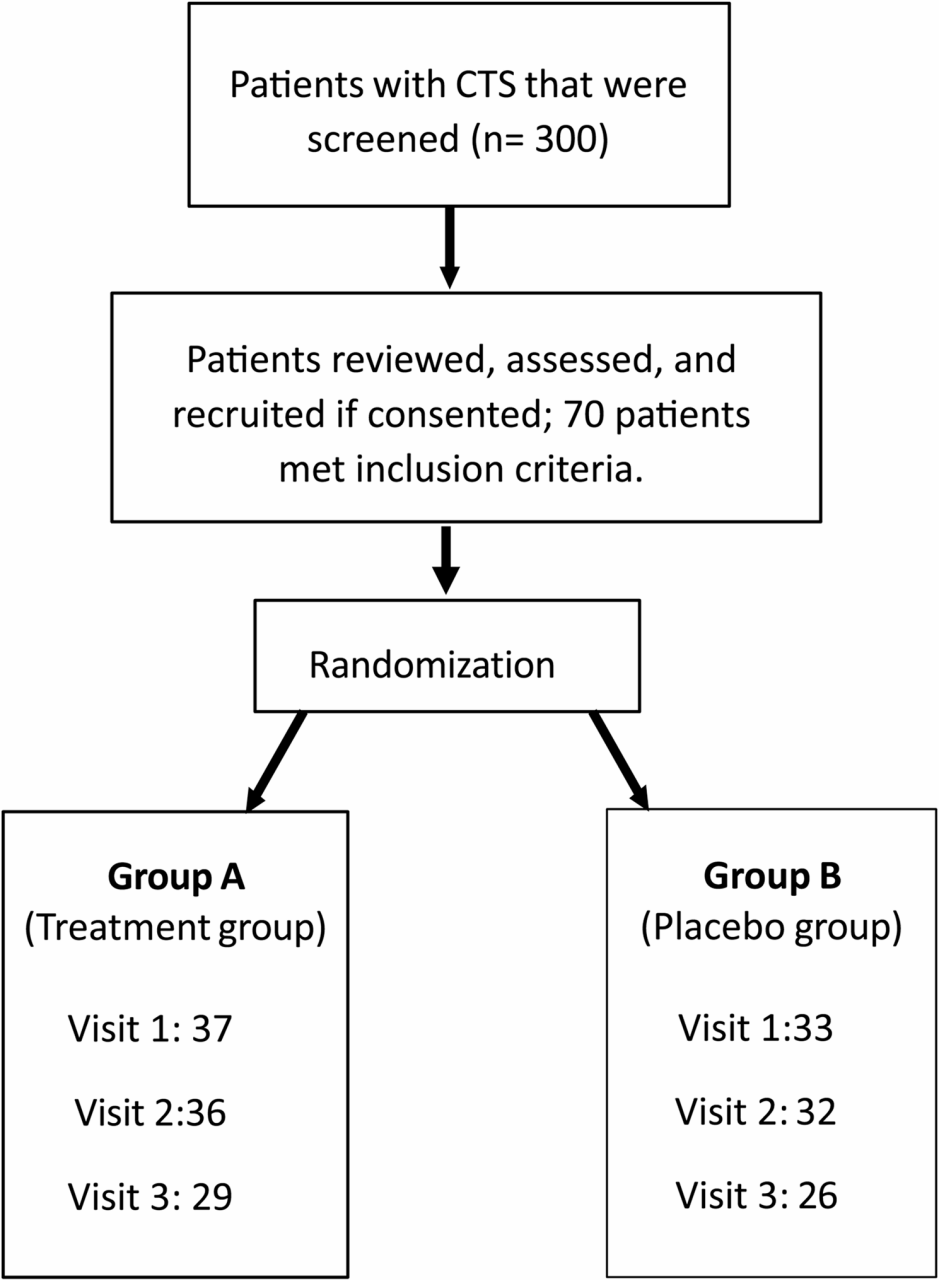
Study flowchart

A total of 70 patients were recruited during the first visit. Of these, 37 patients were assigned to Group A (treatment group) and 33 to Group B (placebo group). During visit 2, a total of 68 patients came for evaluation: 36 patients from Group A and 32 from Group B. Two patients were absent due to logistic issues. Despite multiple reminder calls and messages, only 55 patients attended visit 3, 29 patients from Group A and 26 patients from Group B. The study flow chart is shown in Fig. 1.

The baseline demographics and characteristics of patients showed that both groups were well-matched in age, gender, weight, hand involvement, race, and comorbidities (Table 1). The mean age was 50 years. Females were predominant in both groups, 78.4% (Group A) and 66.7% (Group B). Malays were the largest ethnic group in both groups, followed by Indians and Chinese. Most patients were overweight or obese, with mean weight of 74.1 kg (Group A) and 72.8 kg (Group B). Hypertension, diabetes and dyslipidemia were common comorbidities in both groups. Symptoms were mostly bilateral in both groups with moderate CTS being the most prevalent severity. Physiotherapy attendance was generally low (35.1% in Group A, 30.3% in Group B). Treatment compliance was higher in Group A (73%) compared to Group B (60.6%).

**Table 1:** Baseline demographic

Factors	Groups		*P*-value
	A	B	
Sample size (n)	37	33	
Age (mean, years)	50.9	50.8	0.954
Gender	Frequency, n (%)	Frequency, n (%)	
Male	8 (21.6)	11 (33.3)	0.278
Female	29 (78.4)	22 (66.7)	
Weight (mean, kg)	74.1	72.8	0.76
BMI range (kg/m^2^)	Frequency, n (%)	Frequency, n (%)	
18.5–22.9	3 (8.1)	5 (15.2)	
23.0-26.9	12 (32.4)	11 (33.3)	0.715
> 27	22 (59.5)	17 (51.5)	
Race	Frequency, n (%)	Frequency, n (%)	
Malay	33 (89.2)	28 (84.8)	
Indian	3 (8.1)	5 (15.2)	0.863
Chinese	1 (2.7)	0	
Co-morbidities	Frequency, n (%)	Frequency, n (%)	
Hypertension	9 (24.3)	12 (36.4)	0.279
Diabetes	10 (27)	13 (39.4)	0.278
Dyslipidaemia	15 (40.5)	14 (42.4)	0.875
Rheumatoid arthritis	0	2 (6.1)	0.132
Thyroid disorders	2 (5.4)	4 (12.1)	0.323
Hand involvement	Frequency, n (%)	Frequency, n (%)	
Right	3 (8.1)	6 (18.2)	
Left	5 (13.5)	3 (9.1)	0.356
Bilateral	29 (78.4)	24 (72.7)	
Severity	Frequency, n (%)	Frequency, n (%)	
Mild	11 (30)	12 (36)	0.583
Moderate	26 (70)	21 (64)	
Physiotherapy	Frequency, n (%)	Frequency, n (%)	
Yes	13 (35.1)	10 (30.3)	0.673
No	24 (64.9)	23 (69.7)	
Compliance	Frequency, n (%)	Frequency, n (%)	
Yes	27 (73)	20 (60.6)	0.278
No	10 (27)	13 (39.4)	

### Primary outcomes

The primary outcomes for this study are the changes in the NCS findings after the intervention given. There were no significant different in DSL, SNAP, Velocity, DML and CMAP of both hands between the placebo and the treatment group (Table 2). Nevertheless, there were a relatively better improvement of CMAP in left hand among Group A where the subjects were able to achieve an increment of 0.666 mV. The mean was 8.31 mV at Visit 1 and it improve to 8.98 mV in Visit 2. In comparison, Group B only achieved a 0.025 mV of increment.

**Table 2:** Summary of the NCS findings among group A and group B

Dependent variables	Group	Mean ± SD	Mean differences (V3- V1)	P- value (*P* < 0.05)	Partial ETA squared (*n*^2^)
1. Distal Sensory Latency (DSL), right hand (ms)	A	Visit 01: 4.04 ± 0.44	0.006	0.471	0.011
		Visit 02: 4.11 ± 0.12			
		Visit 03: 4.05 ± 0.65			
	B	Visit 01: 4.56 ± 0.65	−0.2768		
		Visit 02: 4.42 ± 0.21			
		Visit 03: 4.28 ± 0.97			
2. Distal Sensory Latency (DSL), left hand (ms)	A	Visit 01: 4.37 ± 0.96	0.038	0.911	0.002
		Visit 02: 4.29 ± 0.97			
		Visit 03: 4.41 ± 0.23			
	B	Visit 01: 3.99 ± 0.25	0.145		
		Visit 02: 4.01 ± 0.55			
		Visit 03: 4.15 ± 0.80			
3. Sensory Nerve Action Potential (SNAP), right hand (µV)	A	Visit 01: 23.49 ± 0.76	5.646	0.353	0.019
		Visit 02: 28.11 ± 0.85			
		Visit 03: 29.14 ± 0.36			
	B	Visit 01: 23.91 ± 0.16	1.664		
		Visit 02: 25.56 ± 0.13			
		Visit 03: 25.58 ± 0.95			
4. Sensory Nerve Action Potential (SNAP), left hand (µV)	A	Visit 01: 28.90 ± 0.64	0.45	0.583	0.011
		Visit 02: 29.29 ± 0.29			
		Visit 03: 29.35 ± 0.43			
	B	Visit 01: 30.18 ± 0.95	−0.449		
		Visit 02: 33.19 ± 0.92			
		Visit 03: 29.73 ± 0.91			
5. Velocity of Right Hand (m/s)	A	Visit 01: 40.55 ± 0.24	3.1724	0.703	0.006
		Visit 02: 41.22 ± 0.26			
		Visit 03: 43.72 ± 0.51			
	B	Visit 01: 38.68 ± 0.97	1.16		
		Visit 02: 39.04 ± 0.33			
		Visit 03: 39.84 ± 0.64			
6. Velocity of Left Hand (m/s)	A	Visit 01: 39.07 ± 0.24	1.214	0.783	0.004
		Visit 02: 39.30 ± 0.27			
		Visit 03: 40.82 ± 0.81			
	B	Visit 01: 41.54 ± 0.83	2.417		
		Visit 02: 41.29 ± 0.70			
		Visit 03: 43.96 ± 0.93			
7. Distal Motor Latency, right hand (ms)	A	Visit 01: 4.39 ± 0.15	−0.385	0.268	0.024
		Visit 02: 4.08 ± 0.37			
		Visit 03: 4.01 ± 0.28			
	B	Visit 01: 4.72 ± 0.37	−0.197		
		Visit 02: 4.73 ± 0.38			
		Visit 03: 4.52 ± 0.18			
8. Distal Motor Latency (DML), left hand (ms)	A	Visit 01: 4.44 ± 0.88	−0.165	0.497	0.013
		Visit 02: 4.38 ± 0.57			
		Visit 03: 4.27 ± 0.56			
	B	Visit 01: 4.36 ± 0.19	−0.187		
		Visit 02: 4.55 ± 0.57			
		Visit 03: 4.17 ± 0.28			
9. Compound Muscle Action Potential (CMAP), Right hand (mV)	A	Visit 01: 8.18 ± 0.64	0.839	0.888	0.002
		Visit 02: 8.79 ± 0.49			
		Visit 03: 9.01 ± 0.63			
	B	Visit 01: 8.57 ± 0.72	0.842		
		Visit 02: 8.94 ± 0.59			
		Visit 03: 9.41 ± 0.14			
10. Compound Muscle Action Potential (CMAP), Left hand (mV)	A	Visit 01: 8.31 ± 0.74	0.666	0.438	0.015
		Visit 02: 8.60 ± 0.36			
		Visit 03: 8.98 ± 0.14			
	B	Visit 01: 8.57 ± 0.50	0.025		
		Visit 02: 9.05 ± 0.27			
		Visit 03: 8.82 ± 0.39			

Another component that showed a relatively a better outcome in NCS is SNAP in both hands. The mean SNAP of right hand in Group A at baseline was 23.50 µV and it increased to 29.14 µV in visit 3, a 5.65 µV difference. While in group B, the mean SNAP of the right only achieved a 1.66 µV of increment. The mean increased from 23.49 µV in Visit 1 to 25.58 µV in Visit 2. Similarly, the mean SNAP of the left hand in group A showed a slight improvement in Visit 2. The mean increased from 28.90 µV at baseline to 29.35 µV in Visit 2. On the other hand, group B show a slight decrement compared to its baseline in Visit 2. The mean decreased from 30.18 µV to 29.73 µV.

Overall, Group A demonstrated a significantly greater reduction in severity at Visit 2 compared to Group B. Specifically, Group A showed a 15% reduction in moderate severity at Visit 2 relative to Visit 1, whereas Group B only achieved a 6% reduction from baseline. Additionally, Group A recorded a higher increase in the proportion of patients with mild CTS, with a 15% increase from baseline, compared to a 6% increase in Group B (Table 3). This suggests that more patients in Group A experienced improved median nerve conduction, as a greater number transitioned from moderate to mild CTS compared to Group B.

**Table 3:** The summary of severity changes of CTS in group A and group B

Group	A		B		*P*-value
Severity	Mild	Moderate	Mild	Moderate	
Visit 1 (n,%)	11 (30%)	26 (70%)	12 (36%)	21 (64%)	0.738
Visit 2 (n,%)	14 (39%)	22 (61%)	9 (28%)	23 (72%)	0.497
Visit 3 (n,%)	13 (45%)	16 (55%)	11 (42%)	15 (58%)	1.000

### Secondary outcomes

#### SF-36 questionnaire score

The SF-36 questionnaire is used to assess the quality of life, consisting of 8 components (Physical Functioning (PF), Role Limitations due to Physical Health, Role Limitations (RL) due to Emotional Problems (RE), energy/fatigue, emotional well-being, social functioning, pain, and general health (GH), where a higher score indicates a better outcome. In this study, there were no significant differences in all these 8 components between the two groups. However, there is a trend showing that Group A (i.e. treatment group) subjects show better improvement in five out of the eight domains: namely PF, RL, RE, emotional and pain (Table 4).

**Table 4:** SF-36 results in visit 1, 2 and 3 for both group A and B

Dependent variables	Group	Mean ± SD	Mean differences (V3-V1)	*P*- value(*p* < 0.05)	Partial ETA squared (n2)
1. Physical functioning (PF)	A	Visit 01: 61.55 ± 4.62	7.24	0.371	0.018
		Visit 02: 65.86 ± 5.30			
		Visit 03: 68.79 ± 4.41			
	B	Visit 01: 67.11 ± 4.87	0.469		
		Visit 02: 70.96 ± 5.32			
		Visit 03: 67.58 ± 4.41			
2. Role of Physical Limitation (RL)	A	Visit 01: 37.93 ± 6.35	23.276	0.09	0.044
		Visit 02: 61.21 ± 5.62			
		Visit 03: 61.21 ± 5.07			
	B	Visit 01: 56.73 ± 5.86	2.885		
		Visit 02: 56.73 ± 4.01			
		Visit 03: 59.62 ± 3.79			
3. Role of Emotional Limitation (RE)	A	Visit 01: 60.92 ± 5.70	19.54	0.273	0.024
		Visit 02: 68.97 ± 3.56			
		Visit 03: 80.46 ± 4.80			
	B	Visit 01: 77.06 ± 4.10	1.141		
		Visit 02: 75.64 ± 3.70			
		Visit 03: 78.20 ± 5.55			
4. Energy	A	Visit 01: 67.24 ± 5.30	6.724	0.812	0.004
		Visit 02: 69.31 ± 6.41			
		Visit 03: 73.97 ± 4.37			
	B	Visit 01: 65.58 ± 5.2	7.692		
		Visit 02: 70.77 ± 6.35			
		Visit 03: 73.27 ± 4.82			
5. Emotional	A	Visit 01: 77.59 ± 7.74	7.752	0.417	0.016
		Visit 02: 80.04 ± 8.58			
		Visit 03: 84.58 ± 8.01			
	B	Visit 01: 77.23 ± 7.72	3.077		
		Visit 02: 75.69 ± 7.12			
		Visit 03: 80.31 ± 8.23			
6. Social	A	Visit 01: 77.59 ± 4.87	5.604	0.625	0.09
		Visit 02: 77.59 ± 7.76			
		Visit 03: 83.19 ± 6.66			
	B	Visit 01: 72.60 ± 5.99	10.577		
		Visit 02: 77.40 ± 3.78			
		Visit 03: 83.17 ± 3.78			
7. Pain	A	Visit 01: 53.62 ± 7.19	12.586	0.252	0.026
		Visit 02: 62.41 ± 9.46			
		Visit 03: 66.21 ± 9.82			
	B	Visit 01: 63.94 ± 6.92	2.404		
		Visit 02: 67.12 ± 8.96			
		Visit 03: 66.35 ± 7.64			
8. General health (GH)	A	Visit 01: 61.72 ± 3.29	6.207	0.138	0.138
		Visit 02: 61.21 ± 7.33			
		Visit 03: 67.91 ± 6.01			
	B	Visit 01: 54.23 ± 6.43	8.846		
		Visit 02: 62.50 ± 3.10			
		Visit 03: 63.07 ± 7.85			

In the PF domain for instance, Group A achieved a 7.24 increment as compared to Group B which shows deterioration among the three visits, although it was not statistically significant. Similarly in the RL domain, Group A showed an increment of 23.27 while group B showed only 2.88, however it did not reach statistical significance. In the RE and emotional domain, again there was a trend showing increment in Group A versus Group B (19.54 vs. 1.14 and 7.75 vs. 3.07) but this increment was not statistically significant. Another component that showed improvement among the Group A is the pain component where there is a marked increment of 12.58 points from its baseline as compared to Group B which showed a modest increment of 2.40 from its baseline, however it was not statistically significant.

#### BCTQ questionnaire score

Overall, Group A has slightly better improvement as compared to Group B in both components of the BCTQ questionnaire, however they did not reach statistical significance. The mean SSS in Group A decrease by 0.68 points, while Group B showed a smaller reduction by 0.49 points. In terms of FSS component, the mean of FSS in group A had a decrement of 0.45 points from its baseline, while Group B only achieved a reduction of 0.19 points (Table 5).

**Table 5:** BCTQ and VAS score in in visit 1, 2 and 3 for group A and B

Dependent variables	Group	Mean ± SD	Mean differences (V3-V1)	*P*- value(*p* < 0.05)	Partial ETA squared (*n*^2^)
**BCTQ** 1. Functional Status Scale (FSS)	A	Visit 01: 2.30 ± 0.24	−0.456	0.47	0.013
		Visit 02: 1.91 ± 0.39			
		Visit 03: 1.82 ± 0.33			
	B	Visit 01: 1.80 ± 0.30	−0.197		
		Visit 02: 1.72 ± 0.19			
		Visit 03: 1.60 ± 0.19			
2. Symptom Specific Scale (SSS)	A	Visit 01: 2.60 ± 0.16	−0.68	0.451	0.015
		Visit 02: 2.05 ± 0.37			
		Visit 03: 1.92 ± 0.30			
	B	Visit 01: 2.43 ± 0.33	−0.49		
		Visit 02: 1.94 + 0.16			
		Visit 03: 1.94 ± 0.39			
Visual Analogue Scale (VAS)	A	Visit 01: 3.48 ± 0.36	−1.414	0.003	0.103
		Visit 02: 2.79 ± 0.20			
		Visit 03: 2.07 ± 0.20			
	B	Visit 01: 2.81 ± 0.20	0.5		
		Visit 02: 2.31 ± 0.23			
		Visit 03: 3.31 ± 0.28			

#### VAS score

**Fig. 2:**
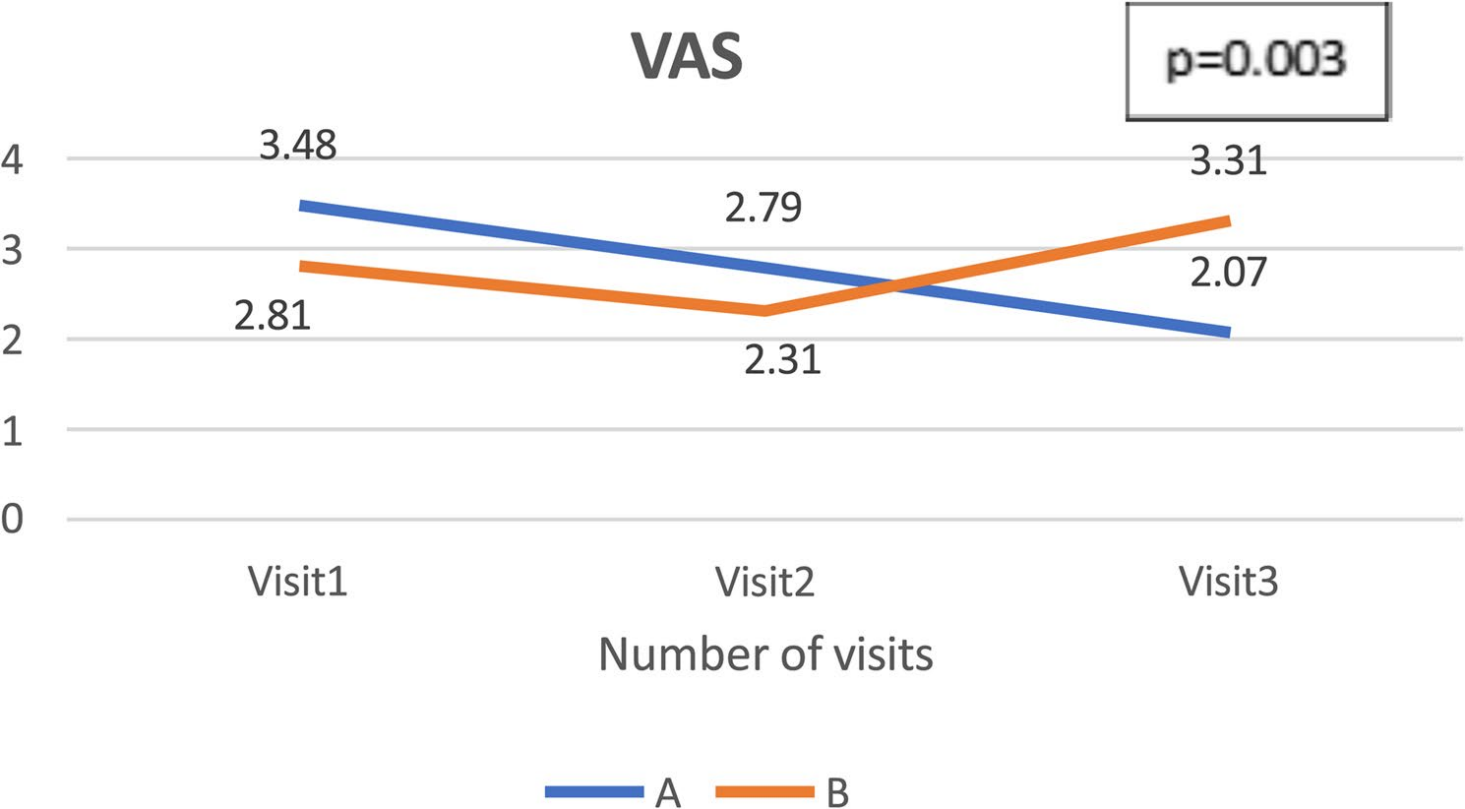
Mean of VAS in Visit 1, 2 and 3 for Group** A** and **B**

The mean VAS score in Group A decreased from 3.48 at baseline to 2.06, while in Group B, it increased from 2.80 to 3.30 (Table 5; Fig. 2). This change was statistically significant with a moderate effect size (*p* = 0.003, η² = 0.103).

#### Tolerability

A higher proportion of participants in Group B reported no side effects compared to Group A (82% vs. 68%) (Table 6). Tolerability in this study was assessed based on patient-reported side effects; however, a definitive causal relationship with the investigational treatment could not be established. Further analyses, such as blood investigations, would be required to determine any potential association.

**Table 6:** Tolerability of treatment drugs

Side effects	Group A (*n* = 37) *n* (%)	Group B *n* (%)	*P*-value
Diarrhoea	5 (13.5)	3 (9.1)	0.866
Abdominal pain	2 (5.4)	0	0.180
Urticaria	1 (2.7)	0	0.393
Weight gain	1 (2.7)	3 (9.1)	0.908
Smelly urine	2 (5.4)	0	0.180
Bloated	1 (2.7)	0	0.936
No side effects	25 (67.6)	27 (81.8)	0.994

## Discussion

The study findings indicate that patients receiving the oral combination of alpha-lipoic acid and vitamin B complex (Bionerv) experienced better pain control compared to the placebo group, as demonstrated by improvements in VAS scores. Although there was no statistically significant improvement in the pain component of the SF-36 or the BCTQ, the modest decrease in VAS scores from 3 to 2 may still be clinically relevant in the context of mild CTS. The baseline pain scores were relatively low likely due to the study’s focus on patients with mild to moderate CTS. While patients in the placebo group initially showed pain reduction in the first three months, this improvement was not sustained, with scores returning to 3 at six months, likely due to a placebo effect. These findings suggest that the combination may offer short-term pain relief, but the evidence for consistent and sustained analgesia is limited and requires further study.

The proposed benefits in quality of life (QoL) were not supported by statistically significant improvements in SF-36 or BCTQ scores. Therefore, any observed changes in these domains should be interpreted with caution. However, the improvement in VAS score is due to the analgesic effects of alpha-lipoic acid, that are likely mediated through multiple mechanisms, including attenuation of median nerve inflammation, reduction of oxidative stress and promotion of nerve regeneration [11]. Furthermore, its ability to inhibit neuronal T-type calcium channels may contribute to decreased neuronal excitability and pain perception [20]. As these mechanisms primarily influence pain modulation, the explanation above has been repositioned to align with pain-related outcomes rather than QoL improvements.

In addition to pain reduction, the oral combination of alpha-lipoic acid and vitamin B complex may contribute to improved quality of life and functional outcomes. Although the observed changes in SF-36 and BCTQ scores were not statistically significant, a trend toward improvement was noted in the treatment group at six months. This may be attributed to the analgesic effects of alpha-lipoic acid, which reduce pain intensity and enable greater engagement in daily activities, ultimately enhancing physical and emotional well-being. Beyond pain relief, alpha-lipoic acid may enhance nerve repair and reduce oxidative stress, potentially improving function and perceived quality of life despite the lack of statistical significance.

The findings of this study are consistent with Passiatore et al., which showed that Alpha Lipoic Acid-R (ALA-R) 600 mg significantly reduced daytime and nighttime pain in mild to moderate CTS patients within two months [9]. Similarly, Notarnicola et al., found that oral ALA supplementation effectively reduced pain over six months, with no added benefit from Extracorporeal Shockwave Therapy (ESWT) [21]. These findings further support the potential role of ALA in CTS management.

Although there was no statistical significance observed in the SSS and FSS components of the BCTQ, there was a larger mean reduction in in both of components in the treatment group, suggesting a possible trend towards symptom control and functional improvement. Our results resonated with several other studies that reported no significant BCTQ differences between treatment and placebo groups [9, 22]. The lack of significant changes in BCTQ scores among ALA-treated CTS patients may stem from mild disease severity where baseline functional impairment may not have been severe enough to detect substantial improvements in BCTQ scores.

- Hence, the use of NCS was necessary to look at objective improvement in CTS severity.

Despite no significant differences in DSL, SNAP, velocity, DML, or CMAP between the placebo and treatment groups, both groups showed mild improvements in electrophysiological parameters over time. Improvements were observed in mean CMAP over the left hand and SNAP in both hands within the treatment group at six months of treatment. However, since similar improvements were seen in the placebo group, the findings may reflect the natural course of recovery or placebo effect rather than a specific treatment effect. Overall, the NCS findings indicate a shift from moderate to mild CTS over time in both groups, where the proportion of mild cases continued to rise, with slightly greater reductions in moderate CTS in the treatment group. These findings suggest that while ALA may support nerve recovery, further studies are needed to confirm its specific contribution.

The tolerability in this study is based on patient reporting side-effects that developed after taking the medications. There are no blood or radiological investigation involved to confirm its association with the symptoms. In this study, most patients who received treatment and placebo group did not develop any side effects. The most common side- effects of the treatment that are observed in this study is diarrhoea, followed with abdominal pain and changed in urine odour. These symptoms are reported to be temporary, and it subsided after two weeks of discontinuation of the medications. Therefore, in this study, the treatment group are found to be tolerable, in agreement with most international studies ([23, 24]).

## Conclusion

Overall, the oral combination of alpha-lipoic acid and vitamin B complex (Bionerv) demonstrated modest but consistent pain relief over six months, with trends suggesting potential functional and quality of life benefits. While the findings were not statistically significant, they align with previous studies supporting the role of ALA in CTS management. These effects may become more evident with a longer treatment duration. Given its tolerability and observed clinical improvements, this combination may serve as a supportive therapy in patients with mild to moderate CTS while awaiting definitive interventions. The limited sample size and study duration may have constrained the ability to detect stronger effects, highlighting the need for larger, longer-term trials.

The strength of this study lies in its RCT design, with computer-generated randomization, minimizing allocation bias. The double-blind approach ensures unbiased assessment. Additionally, the comparable baseline demographics between groups enhance comparability. The study also integrates objective NCS assessments alongside questionnaires, providing valuable insights into the effects of alpha-lipoic acid and vitamin B complex on median nerve electrophysiology.

This study had several limitations, including a small sample size, single-center design with limited ethnic diversity, and non-compliance influenced by pill burden, fasting, and festive seasons. Confounding factors, such as higher physiotherapy attendance in treatment group and unassessed diabetes control, may have affected outcomes. Despite these, findings suggest that alpha-lipoic acid (600 mg) with vitamin B complex (Bionerv) is a promising adjunct therapy for mild to moderate CTS, with prolonged use potentially enhancing nerve regeneration and quality of life.

Future research should involve a larger, multi-center sample for better statistical reliability and patient representation. A dedicated team should improve compliance through reminders, pill-counting methods, and small incentives to reduce dropout rates. Long-term studies should monitor ALA tolerability and safety, assessing liver, kidney, and potential systemic complications.

## Supplementary Information

Supplementary Material 1.

Supplementary Material 2.

Supplementary Material 3.

Supplementary Material 4.

## Data Availability

The datasets supporting the conclusions of this article are included within the article.

## Abbreviations

ALA: Alpha Lipoic Acid
ANOVA: Analysis of Variance
BCTQ: Boston Carpal Tunnel Questionnaire
BMI: Body Mass Index
CI: Confidence Interval
CMAP: Compound Muscle Action Potential
CSI: Corticosteroid Injection
CTR: Carpal Tunnel Release
CTS: Carpal Tunnel Syndrome
DML: Distal Motor Latency
DSL: Distal Sensory Latency
ES: Effect Size
ESWT: Extracorporeal Shockwave Therapy
FSS: Functional Status Scale
GH: General Health
HSAAS: Hospital Sultan Abdul Aziz Shah
I/R: Ischaemia Reperfusion Injury
N: Number
n2: Partial Effect Size
NCS: Nerve Conduction Study
NS: Nighttime Splint
NSAID: Nonsteroidal Anti-Inflammatory Drug
OR: Odds Ratio
PF: Physical Functioning
PIS: Patient Information Sheet
PRP: Platelet Rich Plasma
RA: Rheumatoid Arthritis
RL: Role of Physical Limitation
RE: Role of Emotion Limitation
RCT: Randomized Controlled Trial
ROS: Reactive Oxygen Stress
SF-36: 36 – Short Form Health Survey
SNAP: Sensory Nerve Action Potential
SSS: Symptoms Specific Scale
VAS: Visual Analog Scale

## Declarations

### Background

Carpal Tunnel Syndrome (CTS) is the most common type of nerve entrapment and can cause significant disability by impairing hand functionality. This study aims to evaluate the efficacy and tolerability of an oral combination of alpha-lipoic acid (ALA) and vitamin B complex in alleviating CTS symptoms and improving the quality of life in patients with mild to moderate CTS.
