# Supplementary material for: Evaluating the efficacy and tolerability of the oral combination of alpha lipoic acid and vitamin B complex preparation in carpal tunnel syndrome: a single center, randomized, double-blind, placebo-controlled trial
Source: BMC Neurol. 2025 Dec 8;26:7. doi: 10.1186/s12883-025-04430-y (PMC12766933; doi:10.1186/s12883-025-04430-y)
Supplement: Supplementary file 2 — Supplementary Material 2. [file 12883_2025_4430_MOESM2_ESM.pdf]

SOAL SELIDIK TEROWONG KARPUS BOSTON

- *MALAY VERSION OF THE BOSTON CARPAL TUNNEL QUESTIONNAIRE* (*M-BCTQ*)

(Abdullah et al., 2019)

**Nama**:	**No. Rujukan**: ....
**Tarikh**:	**Tangan** : ( ) **Kanan** ( ) **Kiri**
**SOALAN YANG BERIKUT MERUJUK KEPADA SIMPTOM ANDA BAGI TEMPOH 24 JAM SEPANJANG DUA MINGGU YANG LEPAS.** (Pilih satu jawapan bagi setiap soalan)	
1) Sejauh manakah rasa sakit pada tangan atau pergelangan tangan yang anda alami semasa waktu malam?<br>1- Saya tidak berasa sakit pada tangan atau pergelangan tangan semasa waktu malam.<br>2- Sedikit sakit semasa waktu malam.<br>3- Sederhana sakit semasa waktu malam.<br>4- Amat sakit semasa waktu malam.<br>5- Teramat sakit semasa waktu malam.	2) Berapa kerapkah sakit pada tangan atau pergelangan tangan membuatkan anda terjaga daripada tidur malam sepanjang dua minggu yang lepas?<br>1- Tidak pernah.<br>2- Sekali.<br>3- Dua hingga tiga kali.<br>4- Empat hingga lima kali.<br>5- Lebih daripada lima kali.
3) Adakah anda biasa mengalami rasa sakit pada tangan atau pergelangan tangan semasa waktu siang?<br>1- Saya tidak pernah berasa sakit pada tangan atau pergelangan tangan semasa waktu siang.<br>2- Sedikit sakit semasa waktu siang.<br>3- Sederhana sakit semasa waktu siang.<br>4- Amat sakit semasa waktu siang.<br>5- Teramat sakit semasa waktu siang.	4) Berapa kerapkah anda mengalami rasa sakit pada tangan atau pergelangan tangan semasa waktu siang?<br>1- Tidak pernah.<br>2- Sekali atau dua kali sehari.<br>3- Tiga hingga lima kali sehari.<br>4- Lebih daripada lima kali sehari.<br>5- Sakit berlarutan sepanjang hari.
5) Secara purata, berapa lamakah rasa sakit yang anda alami semasa waktu siang?<br>1- Saya tidak pernah berasa sakit semasa waktu siang.<br>2- Kurang daripada 10 minit.<br>3- 10 hingga 60 minit.<br>4- Lebih daripada 60 minit.<br>5- Sakit berlarutan sepanjang hari.	6) Adakah anda mengalami rasa kebas (hilang deria rasa) pada tangan?<br>1- Saya tidak pernah berasa kebas pada tangan.<br>2- Sedikit kebas.<br>3- Sederhana kebas.<br>4- Amat kebas.<br>5- Teramat kebas.
7) Adakah anda mengalami rasa lemah pada tangan atau pergelangan tangan?<br>1- Saya tidak pernah berasa lemah pada tangan atau pergelangan tangan.<br>2- Sedikit lemah.<br>3- Sederhana lemah.<br>4- Amat lemah.<br>5- Teramat lemah.	8) Adakah anda mengalami rasa kesemutan pada tangan?<br>1- Saya tidak pernah berasa kesemutan pada tangan.<br>2- Sedikit kesemutan.<br>3- Sederhana kesemutan.<br>4- Amat teruk kesemutan.<br>5- Teramat teruk kesemutan.

9) Sejauh manakah rasa kebas (hilang deria rasa) atau kesemutan yang anda alami semasa waktu malam?

1- Saya tidak pernah berasa kebas atau kesemutan semasa waktu malam.
2- Sedikit kebas.
3- Sederhana kebas.
4- Amat kebas.
5- Teramat kebas.

10) Berapa kerapkah rasa kebas (hilang deria rasa) atau kesemutan pada tangan membuatkan anda terjaga daripada tidur malam sepanjang dua minggu yang lepas?

1- Tidak pernah.
2- Sekali.
3- Dua atau tiga kali.
4- Empat atau lima kali.
5- Lebih daripada lima kali.

11) Adakah anda mengalami kesukaran untuk memegang dan menggunakan objek kecil seperti kunci atau pen?

1- Saya tidak pernah mengalami kesukaran untuk memegang dan menggunakan objek kecil seperti kunci atau pen.
2- Sedikit sukar.
3- Sederhana sukar.
4- Amat sukar.
5- Teramat sukar.

12) PADA HARI-HARI BIASA SEPANJANG DUA MINGGU YANG LEPAS, PERNAHKAH SIMPTOM SAKIT TANGAN DAN PERGELANGAN TANGAN MENYEBABKAN ANDA MENGALAMI APA-APA KESUKARAN UNTUK MELAKUKAN AKTIVITI YANG DISENARAIKAN DI BAWAH?

(Sila bulatkan satu nombor yang paling tepat menggambarkan keupayaan anda untuk melakukan aktiviti tersebut)

Aktiviti	Tidak Sukar	Sedikit Sukar	Sederhana Sukar	Amat Sukar	Tidak boleh langsung melakukan aktiviti kerana simptom pada tangan dan pergelangan tangan
Menulis	1	2	3	4	5
Membutangkan baju	1	2	3	4	5
Memegang buku ketika membaca	1	2	3	4	5
Menggenggam gagang telefon	1	2	3	4	5
Membuka penutup balang	1	2	3	4	5
Membuat kerja rumah	1	2	3	4	5
Membawa beg barang runcit	1	2	3	4	5
Mandi dan memakai baju	1	2	3	4	5

(Abdullah et al., 2019)
