# Supplementary material for: Evaluating the efficacy and tolerability of the oral combination of alpha lipoic acid and vitamin B complex preparation in carpal tunnel syndrome: a single center, randomized, double-blind, placebo-controlled trial
Source: BMC Neurol. 2025 Dec 8;26:7. doi: 10.1186/s12883-025-04430-y (PMC12766933; doi:10.1186/s12883-025-04430-y)
Supplement: Supplementary file 3 — Supplementary Material 3. [file 12883_2025_4430_MOESM3_ESM.pdf]

Patient ID:
Date:
Visit:

Malay SF-36

© Ahmad Farouk Musa *et al* 2018
Monash University Malaysia

1. Secara umumnya, kesihatan anda adalah:

- ○ 1 - Cemerlang
- ○ 2 - Sangat baik
- ○ 3 - Baik
- ○ 4 - Biasa
- ○ 5 – Buruk

---

2. **Berbanding setahun yang lalu**, bagaimana anda menilai kesihatan anda secara umum pada waktu **sekarang**?

- ○ 1 - Lebih baik berbanding setahun yang lalu
- ○ 2 - Agak baik berbanding setahun yang lalu
- ○ 3 - Tiada perbezaan
- ○ 4 - Agak merosot berbanding setahun yang lalu
- ○ 5 - Lebih merosot berbanding setahun yang lalu

Item-item berikut merupakan aktiviti yang anda boleh lakukan pada hari-hari biasa. **Adakah kesihatan anda sekarang** membataskan diri anda daripada melakukan aktiviti-aktiviti tersebut? Jika ya, sejauh mana?

		Ya, sangat terbatas	Ya, terbatas sedikit	Tidak, langsung tidak terbatas
3.	**Aktiviti bersemangat**, seperti berlari, mengangkat objek-objek berat, terlibat dalam sukan yang lasak	☐ 1	☐ 2	☐ 3
4.	**Aktiviti sederhana**, seperti mengalihkan meja, memvakum, bermain boling, ataupun golf	☐ 1	☐ 2	☐ 3
5.	Mengangkat atau mengalihkan barangan dapur	☐ 1	☐ 2	☐ 3
6.	Mendaki **beberapa** tingkat anak tangga	☐ 1	☐ 2	☐ 3

7.	Mendaki **satu** tingkat anak tanggga	☐ 1	☐ 2	☐ 3
8.	Membongkok, melutut, ataupun bersujud	☐ 1	☐ 2	☐ 3
9.	Berjalan **lebih daripada satu batu**	☐ 1	☐ 2	☐ 3
10.	Berjalan **beberapa blok**	☐ 1	☐ 2	☐ 3
11.	Berjalan **satu blok**	☐ 1	☐ 2	☐ 3
12.	Mandi atau memakai pakaian	☐ 1	☐ 2	☐ 3

Selama **4 minggu lepas**, adakah anda mempunyai sebarang masalah berkaitan pekerjaan ataupun aktiviti kebiasaan harian anda **akibat daripada kesihatan fizikal anda**?

		Ya	Tidak
13.	Mengurangkan **jumlah masa** anda bekerja atau melakukan aktiviti yang lain	☐ 1	☐ 2
14.	**Pencapaian kurang** daripada yang anda mahukan.	☐ 1	☐ 2
15.	Terbatas dalam melakukan **kerja tertentu** ataupun aktiviti-aktiviti lain	☐ 1	☐ 2
16.	Menghadapi **kesukaran** untuk melakukan kerja atau aktiviti-aktiviti lain (seperti, memerlukan usaha yang berganda)	☐ 1	☐ 2

Selama **4 minggu lepas**, adakah anda mempunyai sebarang masalah dengan kerja ataupun aktiviti kebiasaan harian anda yang lain **akibat daripada sebarang permasalahan emosi** (seperti perasaan kemurungan ataupun kegelisahan)?

		Ya	Tidak
17.	Mengurangkan **jumlah masa** anda bekerja atau melakukan aktiviti lain anda	☐ 1	☐ 2
18.	**Pencapaian yang kurang** daripada apa yang anda mahukan	☐ 1	☐ 2
19.	**Tidak berhati-hati** melakukan kerja atau sebarang aktiviti seperti mana biasa	☐ 1	☐ 2

20. Selama **4 minggu lepas**, sejauh mana permasalahan kesihatan fizikal dan emosi anda mengganggu

aktiviti sosial biasa anda bersama keluarga, rakan, jiran tetangga, ataupun kumpulan?

- ○ 1 - Tidak sepenuhnya
- ○ 2 – Sedikit
- ○ 3 – Sederhana
- ○ 4 - Agak mengganggu
- ○ 5 - Sangat mengganggu

---

21. Sekuat mana kesakitan **tubuh badan** yang anda alami selama **4 minggu lepas**?

- ○ 1 – Tiada
- ○ 2 - Sangat ringan
- ○ 3 – Ringan
- ○ 4 – Sederhana
- ○ 5 – Kuat
- ○ 6 - Sangat Kuat

---

22. Selama **4 minggu lepas**, sejauh mana kesakitan yang anda alami mengganggu kerja biasa anda (termasuk kerja di luar dan dalam rumah)?

- ○ 1 - Tidak sepenuhnya
- ○ 2 – Sedikit
- ○ 3 – Sederhana
- ○ 4 - Agak mengganggu
- ○ 5 - Sangat mengganggu

Soalan-soalan berikut adalah mengenai perasaan dan hal-hal yang anda lalui **sepanjang 4 minggu lepas**. Bagi setiap soalan, sila kemukakan satu jawapan yang paling hampir dengan apa yang anda rasai.

Berapa kerapkah sepanjang **4 minggu lepas.**

		Sepenuh waktu	Kebanyakan waktu	Sedikit waktu	Seseteng gah waktu	Sekali sekala	Tiada
23.	Apakah anda berasa penuh bersemangat?	☐ 1	☐ 2	☐ 3	☐ 4	☐ 5	☐ 6
24.	Adakah anda menjadi seorang yang sangat gementar?	☐ 1	☐ 2	☐ 3	☐ 4	☐ 5	☐ 6
25.	Adakah anda berasa sangat sedih dan tiada apa yang boleh menggembirakan anda?	☐ 1	☐ 2	☐ 3	☐ 4	☐ 5	☐ 6

Patient ID:
Date:
Visit:

26.	Adakah anda berasa tenang dan aman?	☐ 1	☐ 2	☐ 3	☐ 4	☐ 5	☐ 6
27.	Adakah anda bertenaga?	☐ 1	☐ 2	☐ 3	☐ 4	☐ 5	☐ 6
28.	Adakah anda berasa putus asa dan kecewa?	☐ 1	☐ 2	☐ 3	☐ 4	☐ 5	☐ 6
29.	Adakah anda berasa kehilangan segala tenaga?	☐ 1	☐ 2	☐ 3	☐ 4	☐ 5	☐ 6
30.	Adakah anda seorang yang gembira?	☐ 1	☐ 2	☐ 3	☐ 4	☐ 5	☐ 6
31.	Adakah anda berasa penat?	☐ 1	☐ 2	☐ 3	☐ 4	☐ 5	☐ 6

---

32. Selama **4 minggu lepas**, berapa kerapkah **masalah kesihatan fizikal dan emosi anda** mengganggu aktiviti sosial anda (seperti berkunjungan dengan rakan, sanak saudara, dan sebagainya?)

○ 1 - Setiap waktu
○ 2 - Kebanyakan waktu
○ 3 - Sesetengah waktu
○ 4 - Sedikit waktu
○ 5- Tiada

Sejauh mana setiap pernyataan di bawah **BETUL** atau **SALAH** mengenai anda.

		Memang betul	Kebanyakannya betul	Tidak tahu	Kebanyakannya salah	Sangat salah
33.	Saya agak mudah sakit berbanding dengan orang lain.	☐ 1	☐ 2	☐ 3	☐ 4	☐ 5
34.	Saya sesihat seperti sesiapa yang saya ketahui.	☐ 1	☐ 2	☐ 3	☐ 4	☐ 5
35.	Saya menjangkakan kesihatan saya terus merosot.	☐ 1	☐ 2	☐ 3	☐ 4	☐ 5
36.	Kesihatan saya adalah cemerlang.	☐ 1	☐ 2	☐ 3	☐ 4	☐ 5
